# Influence of a revision course and the gender of examiners on the grades of the final ENT exam – a retrospective review of 3961 exams 

**DOI:** 10.3205/zma000980

**Published:** 2015-10-15

**Authors:** Matthäus C. Grasl, Rudolf Seemann, Michael Hanisch, Gregor Heiduschka, Karl Kremser, Dietmar Thurnher

**Affiliations:** 1 Medical University of Vienna, Department of Ear, Nose and Throat Diseases, Vienna, Austria; 2 Medical University of Vienna, Department of Cranio-, Maxillofacial and Oral Surgery, Vienna, Austria; 3 Medical University of Vienna, Department for Medical Education, Vienna, Austria

**Keywords:** Revision course, oral exam, grading, examiner, Medical School

## Abstract

Revision courses should repeat already acquired knowledge and skills and mostly provide a basis for passing the following exam.

**Aim: **The aim of the study is to investigate the influence of a previously attended revision course on the grades achieved in a final exam (Ear, Nose and Throat Diseases). Additionally we ask the question whether the gender of the examiners plays a role concerning the marks or not.

**Methods: **3961 exams at the Department of Ear, Nose and Throat (ENT) Diseases in Vienna were investigated, 725 with revision course (experimental group) and 3236 without previous revision course (comparison group). The revision courses were performed in a standardized way concerning form and content, interactive and case based.

Both groups were examined uniform in regard to topics and time duration. 16 male and 6 female examiners were involved. The grading followed a five–level scale. The examination marks were calculated in the arithmetic mean and median value for the entire sample, gender dependence was calculated according to the Wilcoxon-Mann-Whitney-Test. The inferential statistics included single- and multiple factorial analyses of variance as well as uni- and multivariate regression models.

**Results: **The experimental group achieved a grade average of 2.54 compared with 2.46 for the comparison group. Splitting up into male and female examiners, an average of 2.54 and 2.58 resp. for the experimental group and 2.44 and 2.61 resp. for the comparison group resulted. Female examiner marked significantly lower grades in comparison to their male colleagues (P= 0.001926).

**Conclusions: **The ENT revision course did not improve the grade averages of the final ENT exam. Female examiners grade stricter than male examiners. There was no difference concerning grades 4 (pass) and 5 (fail) but female examiners grade less with mark 1.

## Zusammenfassung

Repetitorien sind Lehrveranstaltungen, die bereits erworbenes Wissen und Kenntnisse wiederholen und meist als Vorbereitung zum Bestehen eines Examens dienen.

**Zielsetzung: **Zweck der Studie ist es, die Beeinflussung des Ergebnisses bei der mündlichen Gesamtprüfung (Rigorosum) im Fach Hals-Nasen-Ohrenheilkunde zu untersuchen, wenn zuvor ein interaktives mündliches Repetitorium besucht wurde. Zudem wird der Frage nachgegangen, ob es Unterschiede zwischen weiblichen und männlichen Prüfern bei der Notenvergabe gibt.

**Methode:** 3961 Einzelprüfungen an der Universitätsklinik für HNO-Krankheiten in Wien wurden untersucht, 725 mit Repetitorium (Versuchsgruppe) und 3236 ohne Repetitorium (Vergleichsgruppe). Das Repetitorium wurde als inhaltlich standardisiertes Wahlfach interaktiv und fallorientiert gestaltet.

Beide Gruppen wurden nach einheitlichen Kriterien (Inhalte, Dauer) geprüft. 16 männliche Prüfer und 6 weibliche Prüferinnen waren tätig. Die Benotung erfolgte in einer 5-teiligen Skala und wurde als arithmetischer Durchschnittswert mit Median, die Geschlechtsabhängigkeit der Notenvergabe wurde mit dem Wilcoxon-Rangsummentest berechnet. Die schließende Statistik bestand aus ein- und multifaktoriellen Varianzanalysen sowie uni- und multivarianten Regressionsmodellen.

**Ergebnisse: **Die Versuchsgruppe erzielte einen Notendurchschnitt von 2,54 gegenüber jener der Vergleichsgruppe mit 2,46. Nach Aufteilung in männliche und weibliche Prüfer ergibt sich ein Notendurchschnitt für die Versuchsgruppe von 2,54 bzw. 2,58 und für die Vergleichsgruppe von 2,44 bzw. 2,61. Der Unterschied ist statistisch signifikant (p=0,001926).

**Schlussfolgerung: **Das HNO-Repetitorium hat nicht zu einer Verbesserung des Notendurchschnittes bei der nachfolgenden mündlichen Prüfung über Inhalte des gesamten Faches geführt. Weibliche Prüfer benoten strenger als männliche. Der wesentliche Unterschied zwischen männlichen und weiblichen PrüferInnen in der Notengebung besteht bei der Vergabe der Noten 1 und 2 und nicht bei der Note 5 (nicht bestanden).

## 1. Introduction

### 1.1 Background

In revision courses, students and/or co-workers are being taught selected cognitive and procedural knowledge within a few days or weeks [1]. Focused selection and presentation of topics relevant for the exam are important. Knowingly omitting or emphasizing topics are challenges, which essentially influence the outcome of a revision course [2]. In the Anglo-American regions, revision courses are part of the regular curriculum [3], [4], [5], [6], [7], [8], [9]. In German speaking regions they are mostly optional courses [10], [11], [12], [1]. Most of the studies deal with less than 100 exams and survey periods of mostly one year. Survey periods of 5 years and student numbers of several thousand are missing completely. At the Medical University of Vienna, the curriculum committee recommended optional revision courses to complete the syllabus to prevent delays of students to pass the exams [13]. Similar courses are being offered commercially by private organizations at high costs. The final ENT exam is an oral general exam including cognitive and procedural knowledge about the most frequent and most threatening diseases. 

#### 1.2 Aim of the study

The focused transfer of knowledge in presence based revision courses tries to compensate for knowledge gaps by intensive repetition of subjects. Several authors state improvement of exam results caused by the attendance of revision courses [14], [2], [6], [10], [11].

The relevance of our Study “Influence of a revision course and the gender of the examiners on the grades of the final ENT exam – a retrospective review of 3961 exams” is given by the fact that we want to test the generally valid opinion that revision courses improve the results of following exams. 

A possible bias caused by examiners is a frequent risk at exams. Especially the gender bias of examiners at oral exams remains unclear. So far, the international literature shows different results. There is no agreement that examiners prefer candidates of their own gender [15]. Male examiners are not more stringent than female examiners [16]. Male examiners grade female students significantly better than male students [12].

This study especially addresses factors of program evaluation and thus is of general value. It includes understanding, confirmation and improvement of effects of intervention. The study tests whether we do what we think or not. A target-aimed support should result. Publication of our data should serve in comparison with other programs as basis for the decision to make changes or not. 

The aim of the study is to test the influence on the results of the ENT final exam after participation in an interactive oral revision course. Additionally we ask the question, whether there are differences between male and female examiners concerning the grading or not. 

## 2. Methods

### 2.1 Background

The curricular regulation for the doctoral viva („Rigorosenordnung“) was issued for the medical schools in Austria at the beginning of the 20^th^ century and remained with few changes in effect until the new curriculum (MCW, Medical Curriculum Vienna) was introduced in 2002. The students had to pass oral exams in all relevant disciplines [17]. The final exam for ENT was introduced in 1979 in Vienna Medical School.

#### 2.2 Study design

The present study is a retrospective analysis of the grades of the final ENT exam in the 3^rd^ section of the medical study at the Medical University of Vienna. A part of the student population has attended the revision course prior to the final ENT exam.

#### 2.3 Description of sample 

The administrator for student’s affairs at the Department for ENT has digitally recorded grading results of 19260 exams between Oct. 1^st^, 1997 and Jan. 31^st^, 2013 without personal reference to the students examined. Additionally he coded and recorded data of the examiners, their gender, students and their gender. Thus, the basic set of data could be used in an anonymous way. Data of about 10% of the students who repeated the exam were recorded additionally, but not analyzed in this study.

For this study, between Oct. 1^st,^ 1997 and Jan. 31^st^, 2009, data from the revision courses and data of 3961 exams were included. 16 male and 6 female examiners were active. The gender of the students was included as a variable. 

#### 2.4 Framework conditions of the final exam in the discipline ENT and the parallel revision course

4 hours per week credit hours lecture and 1 credit hour practical course were designed for the discipline ENT. During the period of our investigation the revision course was offered additionally at no charge as an enhacement of the syllabus as a preparation for the final ENT exam. This elective course “System oriented revision course in ENT” was offered ongoing by one teacher and included 7 topics of complexes of symptoms. Based on clinical symptoms, interactively in a “question-answer-format” the revision of the topics was symptom and case oriented. At the beginning of each unit, the teacher informed the students about that the revision course would cover the syllabus of the system complexes (e.g. breathlessness, sore throat, etc.) but not the whole syllabus for the final ENT exam, which was listed in a catalogue for both students and teachers. The syllabus of the revision course covered 50-60% of the syllabus for the final ENT exam. Thus, the topics for the ENT exam were standardized. Also a mandatory minimum examination time of 15 minutes was specified. The students could normally choose an examiner. 

The grading scale for the final exam was a 5-part scale: 1=excellent, 2=good, 3=satisfactory, 4=sufficient, 5=not sufficient. 1-4 meant passed, 5 meant failed and the exam needed to be repeated. 

A second reviewer guaranteed the objectivity of evaluation and interpretation of the measuring instrument final ENT exam. A second examiner could have improved the objectivity of the performance, but sufficient resources were not available. The examiner takes part in the exam, which results in a low reliability [18] and is reason for low validity [19]. The revision course was not evaluated.

#### 2.5 Statistical analysis

Data analysis was performed using the „Statistical Package for the Social Sciences – SPSS“ version 20.0 for Windows. The examination marks were calculated in the arithmetic mean and median value for the entire sample, for the experimental group with revision course, for the comparison group without revision course as well as in all groups according to the gender of the examiner. Moreover the percentage of the particular examination marks of oral examinations was quantified totally and in terms of gender of the examiners. The impact of the factors revision course (yes/no), gender of the examiner (female vs. male) and the gender of the students (female vs. male) were determined by means of single factor analysis of variance, recoding the gender for the calculation. Taking into account the multiple testing all these three factors were calculated repeatedly in a multifactorial analysis of variance. Furthermore, the impact on the rating was described by linear regression analysis. The inferential statistics consisting of single- and multiple factorial analyses of variance as well as uni- and multivariate regression models were carried out with the open source statistical program „R version 3.1.2“ [https://www.r-project.org/].

## 3. Results

### 3.1 Participants

We analyzed 3961 exams from a single institution, the department of ENT at the Medical University of Vienna, wherefrom all examiners came.

#### 3.2 Results of the exams 

Table 1 (Tab. 1) shows the results for all examiners according to the gender of the examiners for the experimental group (with revision course) and comparison group (without previous revision course).

##### 3.2.1 Average grade in the overall collective

In the overall collective of the examiners the experimental group with revision course (n=725; Grade: 2.54±1.38) was graded in poor direction compared to the comparison group without revision course (n=3236; Grade 2.46±1.34). When used in analysis of single factor variance statistically no significant results were certifiable (ANOVA: Df=1; square sum=4.2; root mean square=4.1559, F=2.2865; P=0.1306). In the univariate linear regression resulted a statistically not significant effect of 0.08376 (P=.131 by Wald test) for lower grades of students which completed the revision course.

##### 3.2.2 Dependency of the results of the exams on examiners gender

The examination grades of male examiners were on average 2.45 with a standard deviation of 1.36 and a sample size of 3376. The examination marks of female examiners were on average 2.60 with a standard deviation of 1.28 and a sample size of 585. Female examiner marked significantly poorer grades in comparison to their male colleagues (Wilcoxon rank sum test: W=910610, P=.001926).

##### 3.2.3 Dependency of the results of the exams of student’s gender

The arithmetic mean of the examination grades of female students were 2.45±1.34 and 2.51±1.36 for their male colleagues, respectively. In the single factorial analysis of variance, students gender proved not to be a significant influencing factor (ANOVA: Df=1; square sum=3.6; root mean square=3.64; F=2.0028; P=0.1571). In the analogous single variant regression model the effect of female was 0.06233 on the average grade with no significance (P=.157 by Wald test).

##### 3.3.4 Multifactorial analysis of variance

Taking into account the multiple testing, the three factors revision course, gender of the examiner and the gender of the students were tested in a multivariate regression analysis (see Table 2 (Tab. 2)), resp. a multifactorial analysis of variance (see Table 3 (Tab. 3)). Only the gender of the examiners proved to be furthermore a significant factor of influence. On the average, female examiners assigned 0.146 points lower grades.

##### 3.2.5 Average grades, number and percent of fails (“not sufficient”) compared with the gender of students

There is a special emphasis on grade 5 (not sufficient). The percentage varied between 12.23% for the combination female candidate/female examiner and 8.13% percent for the combination male candidate/female examiner (see Table 4 (Tab. 4)).

The average over all combinations is 10.2% and thus showed a very homogenous grading for the decision pass or fail.

## 4. Discussion

### 4.1.Value of the results and comparison with the existing literature

In the overall collective of the examiners the experimental group with revision course was graded in poor direction compared to the comparison group without revision course. When used in analysis of single factor variance, statistically no significant results were found, and in the univariate linear regression there resulted a statistically not significant effect for lower grades of students who completed the revision course. 

Literature shows high efficiency and acceptance by the students for revision courses. On the other hand, the effort, which is balanced by a good preparation for exams or for the job, should not be underestimated [2]. 

Throughout, revision courses can be seen as useful courses in different forms. Students state that their knowledge is better [6] or even significantly better [14] than before and a revision course is effective as a preparation for state exams [10]. Students are no longer anxious but motivated for further independent learning. Grades improved and significantly less students fail the following exam. The limited period guarantees continuous learning. Weak and good students benefit likewise [1]. Revision courses are efficient, cost-effective and adaptable for high numbers of students [3]. For tests of practical skills performance and success improve [5].

Our results are contrary to relevant literature.

Female students showed an average grade of 2.45±1.34, their male colleagues 2.51±1.36. In the single factor analysis of variance student's gender proved not to be a significant influential factor. In the univariate linear regression model the effect of female gender with -0.06233 at the average of marks was classified as not significant. 

These results comply with literature [12], [15], [16], [17], [18].

#### 4.2 The ENT revision course did not improve the average grades for the following final oral ENT exam covering the whole subject.

The teacher of the revision course was examiner as well. There was no bias concerning participants of the revision course. The average grades of students having attended the revision course were only marginally better. Differentiation between well and poor performing students was not carried out. 

Why, contrary to our assumption, students having attended a revision course compared with students not having attended a revision course did not perform better but even slightly poorer, can be explained as follows: students think that the revision course is a substitute for self-studies and not a supplement and revision and a time saving way. The final ENT exam is one of the last exams of their medical studies. Additionally there could have been a longer time span between the revision course and the exam and the revision course only covered 50-60% of the topics examined in the final exam. It cannot be totally dismissed, that the revision course possibly promoted the effect of “bulimic learning”, which means learning for a single test, keeping knowledge in the short time memory, spitting it out at the exam without digestion. We did not ask the students for their motivation to attend the revision course. Possible reasons are:

- Offer to attend a tight teaching/learning program
- Interest in ENT topics
- Structured interactive and case oriented tuition by a teacher with high subject-specific and didactic competence
- Compensation of the missing involvement with the subject
- “Distance learning” without attending lectures
- Promotion of further self-directed learning
- Reduction of fear and uncertainty before the exam

#### 4.3 The average examination grades of female examiners are significantly poorer than those of male examiners

Bias of exam results caused by examiners are a latent risk at exams. Humans behave differently and it is not surprising that examiners show tendencies of preference and discrimination of candidates. Knowing this, measures should be taken as early as possible. These vary from randomized candidate distribution or a board of examiners or even changing from an oral to a written exam [20]. Different features of the candidates, not yet examined, could cause a possible reason for gender dependent grading. These variables could influence, to a different extent, male and female examiners, which makes it necessary to explain different connections [15]. Free choice of examiners, age of candidates, experience of examiners and sympathy or reservation regarding the candidate. Wiskin et al. [21] describe in their paper about gender as a variable at the OSCE about communication at the level of the last year of studies, that female examiners give better grades. They state that this could be prevented by a strategic gender equality for men and women. Also Boehm et al. [12] report a better grading by female examiners in the final exam Social Medicine. McManus et al. [22] propose to solve the problem of a bias caused by gender or ethnic origin at practical OSCE stations, by deploying 2 examiners per station. Using multifactorial Rasch modelling they estimate the effect that examiners prefer or aggravate, and compensate the effect by pairing toughest and least tough examiners. 

Despite the possible bias, all our examiners valued this kind of exam. It offered the possibility to examine declarative and procedural knowledge case and symptom oriented together with basic knowledge. Still, there was no significant difference for the fail ratio between male and female examiners.

#### 4.4 Strengths and weaknesses of the study

##### 4.4.1 Strengths

The authors consider the clear concept of this retrospective study as strength. The high number of cases allow clear statistical statements. During the long data collection period, 22 examiners were included.

##### 4.4.2 Weaknesses

The design is simple and not very robust, but easily done and cost saving. There was no structural feedback from students and examiners, which could have given insight into individual-related bias caused by the interaction between examiners and candidates. We have no data about how often Students attended the revision course and how close the attendance was prior to the exam. We did not consider especially the repeaters (10%) in our calculations. Having performed an evaluation of the revision course, we could have distinguished between “reaction level” and “learning level” [23], [24]. 

## 5. Conclusions

We conducted this study to find out the advantage and the influence of the gender of examiners of the final ENT exam at the Medical University of Vienna. The ENT revision course did not improve the average grades at the final ENT exam. The average grades of female examiners were significantly worse than the grades of male examiners. The main difference between the grading of female and male examiners was for grades 1 and 2 and not for grade 5 (fail). All examiners were very close for the decision between 4 (pass) and 5 (fail). As a relative limit of this study, a low reliability can be seen which also causes a low validity. After the introduction of a new medical curriculum in 2002, which does not include final ENT exams any more, also the revision courses for the final ENT exam were discontinued. However, in the first year of the new curriculum several teachers offer optional revision courses close to the exam at the end of the school year, without registration.

## Acknowledgements

The authors give thanks to all teachers of the ENT department of the Medical University of Vienna, who participated at this study as examiners. 

## Competing interests

The authors declare that they have no competing interests.

## Figures and Tables

**Table 1:**
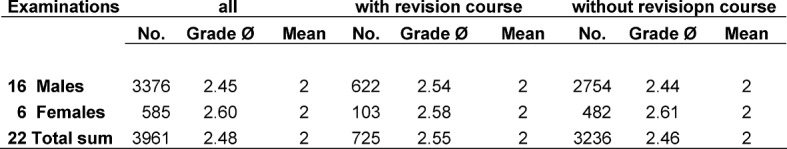
Arithmetic mean and median value of all examination results, from the experimental group with revision course and the comparison group without revision course, separated by the gender of the examiner.

**Table 2:**
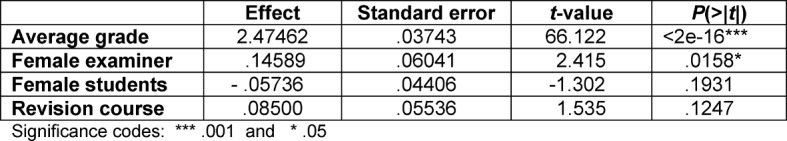
Multivariant regression model of the grading estimated by genders of examiners and students as well as the studets participation in the revision course. The avergae grade corresponds to the reference group of male students examined by male examiners.

**Table 3:**
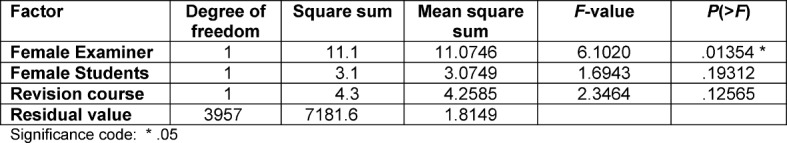
Multifactorial analysis of variance of grading.

**Table 4:**
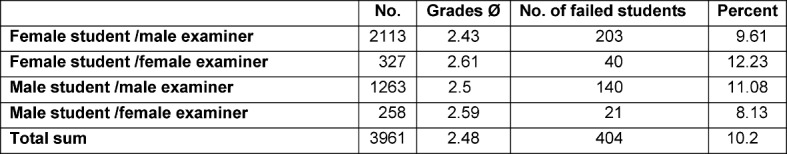
Average grades , number and percentage of failed students compared to gender of students and examiners.

## References

[1] Störmann S, Chiapponi C, op den Winkel M, Wöck M, Bender J, Kern AB, Gebhardt C, Angstwurm M (2010). Mit dem Internet zum Examen - Prüfungsvorbereitung mit dem virtuellen Staatsexamen-Repetitorium der LMU München. http://dx.doi.org/10.3205/10gma143.

[2] Kühn J, Jabs WJ (2007). Das Lübecker Repetitorium "Innere Kompakt": ein Pilotprojekt zur Vorbereitung auf das neue zweite Staatsexamen. GMS Z Med Ausbild.

[3] Hibbert EJ, Lambert T, Carter JN, Learoyd DL, Twigg S, Clarke S (2013). A randomized controlled pilot trial comparing the impact of acces to clinical endocinology video demonstrations with access to usual revision resources on medical student performance of clinical endocrinology skills. BMC Med Educ.

[4] Lymn JS, Mostyn A (2010). Audience response technology: Engaging and empowering non-medical prescribing students in pharmacology learning. BMC Med Educ.

[5] Meade O, Bowskill D, Lymn JS (2009). Pharmacology as a foreign language: A preliminary evaluation of podcasting as a supplementary learning tool for non-medical prescribing students. BMC Med Educ.

[6] Mole G, Gillespie L (2012). Those who can, teach. Accessing medical students`perception of a finals revision programme delivered by foundation and core trainees: a cross-sectional study. BMJ Open.

[7] Freimanis AK (1970). Successful teaching of radiology of medical students and interns. With emphasis on third and fourth-year programs. Radiology.

[8] Swartz TH, Lin JJ (2014). A clinical refresher course for medical scientist trainees. Med Teach.

[9] Brown G, Manogue M (2001). AMEE Medical Education Guide No. 22: Refreshing lecturing: a guide for lecturers. Med Teach.

[10] Rengier F, Rauch PJ, Partovi S, Kirsch J, Nawrotzki R (2010). A three-day anatomy revision course taught by senior peers effectively prepares junior students for their national anatomy exam. Ann Anat.

[11] Bredemeier S, Pabst R, Nave H (2005). Der "Makro-Marathon": Ergebnisse der Evaluation eines Repetitoriums der makroskopischen Anatomie zur Vorbereitung auf das Physikum. GMS Z Med Ausbild.

[12] Boehm G, Bernhard G, Kwizda-Gredler B, Kunze U, Rathmanner T, Rieder A, Schoberberger R, Schwarz B, Vutuc C, Kunze M (2001). Einfluss von Geschlecht und Studiengebühren auf die Noten bei Rigorosum-Prüfungen im Prüfungsfach Sozialmedizin.

[13] Bundesministerium für Wissenschaft und Forschung (1992). Studienplan für die Studieneinrichtung Medizin an der Medizinischen Falkultät der Universität Wien.

[14] Webb AL, Choi S (2014). Interactive radiological anatomy eLearning solution for first year medical students: Development, integration, and impact on learning. Anat Sci Edu.

[15] Denney ML, Freeman A, Wakeford R (2013). MRCGP CSA: are the examiners biased, favouring their own by sex, ethnicity, and degree source?. Br J Gen Pract.

[16] McManus IC, Elder AT, Dacre J (2013). Investigating possible ethnicity and sex bias in clinical examiners: an analysis of data from the MRCP(UK) PACES and nPACES examinations. BMC Med Educ.

[17] Ministerium für Kultus und Unterricht (1899). Das Studium an der medizinischen Fakultät. Die medizinische Rigorosenordnung. Verordnung des Ministers für Kultus und Unterricht vom 21. Dezember 1899, RGBl. Nr. 271.

[18] Davis MH, Karunathilake I (2005). The place of the oral examination in today's assessment systems. Med Teach.

[19] Schuhwirth LW, van der Leuten CP (1996). Qualitiy control: assessment and examinations. Z Hochschuldidaktik.

[20] Niehaus DJ, Jordaan E, Koen L, Mashile M, Mall S (2012). Applicablity and fairness oft he oral examination in undergraduate psychiatry training in South Africa. Afr J Psychiatry (Johannesbg).

[21] Wiskin CM, Allan TF, Skelton JR (2004). Gender as a variable in the assessment of final year degree-level communication skills. Med Educ.

[22] McManus IC, Thompson M, Mollon J (2006). Assessment of examiner leniency and stringency ('hawk-dove effect') in the MRCP(UK) clinical examination (PACES) using multi-facet Rasch modelling. BMC Med Educ.

[23] Jünger J, Just I (2014). Empfehlungen der Gesellschaft für Medizinische Ausbildung und des Medizinischen Fakultätentags für fakultätsinterne Leistungsnachweise während des Studiums der Human-, Zahn- und Tiermedizin. GMS Z Med Ausbild.

[24] Kirkpatrick D (1998). Evaluating Training Programs: The Four Levels.

